# Anatomic Variations of the Perineal Arteries and Nerves in the Male and Female Dog and Its Clinical Implications

**DOI:** 10.3390/ani13121912

**Published:** 2023-06-08

**Authors:** Nieves Martín-Alguacil, Luis Avedillo

**Affiliations:** Departamental Section of Anatomy and Embryology, School of Veterinary Medicine, Universidad Complutense de Madrid, 28040 Madrid, Spain; luiavedi@ucm.es

**Keywords:** perineal vascularization, perineal innervation, anatomical variations, perineal surgery, ischiorectal fossa, anal sphincter, faecal incontinence, dog

## Abstract

**Simple Summary:**

The study of perineal arteries and nerve distribution in dogs has shown that there are several anatomical variations that may be encountered in clinical practice, highlighting the importance of understanding the blood supply and innervation of the perineal region. There is a description of the perineal arteries to be used as a reference, in which the dorsal perineal artery is shorter and only vascularises the dorsal part of the perineum. Whereas in the vascular variations described in this study, the long-type perineal artery and the perineal trunk passed distally beyond the point of origin of the ventral perineal artery. The classic pattern of perineal artery distribution was observed in only 46% of the specimens with most of the remaining cases showing either a dorsal perineal artery “long type” or a perineal trunk variation. The perineal nerve was found to always follow the route of the dorsal perineal artery “long type” and perineal trunk but not when presented in the traditionally described vascular pattern. These findings are important for anatomists, surgeons, and anatomy teachers and students. For the latter, it is an added value, as it is important for them to understand how different anatomical regions can be from anatomy books.

**Abstract:**

The anatomic variations of the perineal arteries and nerves are studied in the dog. The aim of the study is to provide a more detailed understanding of the blood supply and innervation of the perineal region, providing detailed information on the perineal arteries and nerve distribution and their variability in male and female dogs. The study used 232 pelvic halves from 116 adult dogs and analysed the differences using the chi-squared test. The results showed that the presumptive model for perineal artery distribution described in N.A.V. was observed in 46% of the specimens. Additionally, a dorsal perineal artery “long type” was found in 13% of the dogs, and a perineal trunk was present in 41% of the dogs. In the study, there was no variation in perineal nerve distribution, and it was found that the perineal nerve did not run together with the dorsal perineal artery when it was the “short type” as described in the presumptive model for perineal artery distribution. Instead, it always followed the route of the dorsal perineal artery “long type” and the perineal trunk when they were present. The findings of the study may be useful for veterinary surgeons when approaching the perineal region in dogs.

## 1. Introduction

Anatomic variations of perineal arteries in the dog are occasionally noted during routine cadaveric dissections. These anomalous vessels may be at risk during dissections and suturing or any surgical procedure involving the perineal region, and it is important for veterinarians to have a thorough understanding of the potential vascular variations and the precise innervation.

In the traditional anatomical model, the internal iliac artery (IIA) gives rise to the umbilical artery, the internal pudendal artery, and the caudal gluteal artery. The internal pudendal artery branches are the prostatic/vaginal, ventral perineal, and penile/clitoral arteries, and the caudal gluteal artery branches are the iliolumbar, cranial gluteal, lateral caudal, satellite of the ischiatic nerve, and dorsal perineal arteries. However, studies have shown anatomical variations in the caudal gluteal and internal pudendal arteries and their branches [1,2]. To evaluate the presumptive variations of the IIA in dogs, Avedillo et al. [1,2,3] established a model of the general organization and distribution of the canine IIA using the guidelines established by the Nomina Anatomica Veterinaria [4]. The model was based on an “official illustration” made by Paul Simoens [5] and was designed to be easily followed. When the model was compared to their findings, several anatomical variations were described in the caudal gluteal [1] and the internal pudendal [2] arteries and their branches.

There are many surgical procedures where perineal artery branches may be compromised, including anal sacculectomy, perianal mass excision [6,7], perianal fistulectomy [8,9], perineal herniorrhaphy [6], vulvar fold excision, and perineal trauma repair [10,11,12], and may result in post-operative dehiscence and undesirable surgical iatrogenic complications. Faecal incontinence resulting from damage to the external anal sphincter or its innervation may also be a potential complication [6]. On the other hand, successful aesthetic and functional perineal reconstruction requires adequate skin cover and well-vascularised tissue. Various flaps, such as the caudal superficial epigastric, lateral caudal, and deep circumflex iliac axial pattern flaps, are routinely used for cutaneous reconstruction of the perineum [6,12,13,14,15,16,17,18,19]. The scrotal flap [10,20] and dorsal vulvar skin [11] have also been successfully used as a transposition flap for the closure of perineal skin defects in male and female dogs, respectively. It is critical to include and preserve the direct cutaneous vessel in axial pattern flaps.

A thorough understanding of potential vascular variations and the precise innervation in the perineum and the external anal sphincter is essential for veterinarians to minimize the risk of post-operative complications and achieve successful surgical outcomes. The purpose of this research is to explore the anatomical and clinical significance of vascular anomalies related to the perineal arteries and their relation to nerve disposition.

## 2. Materials and Methods

The 122 dogs used in this study came from dissecting and post-mortem rooms. The dogs used were legally procured in accordance with the regulations and laws of the European Union (86/609/EEC) and Spain (RD 223/1998) for the care, use, and housing of animals in research. The study was approved by the Complutense University of Madrid Bioethics Committee, indicating that ethical considerations were taken into account.

Due to the large number of cross breeds, the dogs were grouped into three categories based on head shape or weight. The specimens were prepared and injected with coloured natural latex to aid the identification of the arteries and nerves. The IIA and its major branches were exposed, and photographs and drawings were made systematically. During the entire process of dissection, special attention was paid to the origin and variations of the perineal arteries and perineal nerves. The data collected during the dissection process were recorded for further analysis.

The chi-squared test for independence or homogeneity was used to analyse the differences between gender and side, and the results were considered statistically significant if *p* ≤ 0.05 (Table 1). This statistical analysis helped to determine the relationship between different variables, such as the frequency of specific variations in the perineal arteries and perineal nerves.

## 3. Results

The statistical study examined 232 hemi-pelvises belonging to 116 adult dogs with an equal distribution of males and females. The number of dogs in each category was as follows: (1) head shape: 15 brachycephalic, 90 mesaticephalic, and 11 dolichocephalic dogs; and (2) weight: 31 small (<6 kg), 23 medium (7–20 kg), and 62 large (>20 kg) dogs.

**Figure 1:**
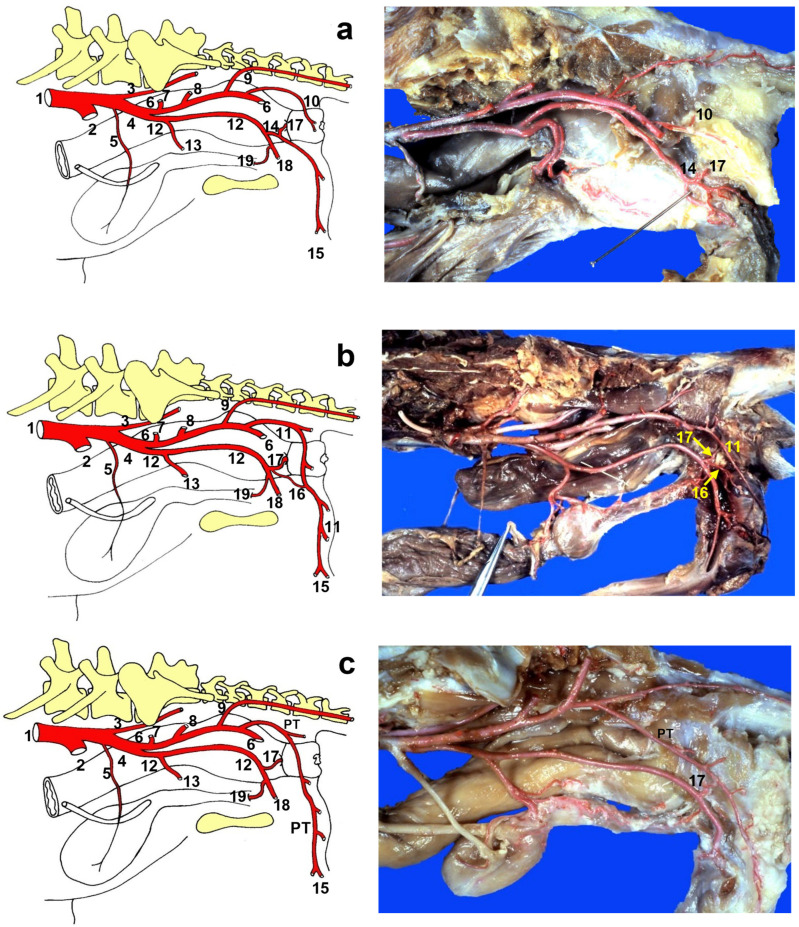
Schematic representation and dissection of the anatomical variations of the perineal arteries in the dog (lateral view). (**a**) NAV vascular pattern, dorsal perineal artery short type; (**b**) dorsal perineal artery “long type”; and (**c**) perineal trunk. 1: Abdominal aorta; 2: External iliac artery; 3: Median sacral artery; 4: Internal iliac artery; 5: Umbilical artery; 6: Caudal gluteal artery; 7: Iliolumbar artery; 8: Cranial gluteal artery; 9: Lateral caudal artery; 10: Dorsal perineal artery short type; 11: Dorsal perineal artery long type; 12: Internal pudendal artery; 13: Prostatic/vaginal artery; 14: Ventral perineal artery; 15: Dorsal scrotal branch/dorsal labial branch; 16: Minor ventral perineal artery; 17: Caudal rectal artery; 18: Artery of the penis/clitoris; 19: Urethral branch. PT: Perineal trunk.

The classic vascular model used for comparisons (Figure 1a) had a shorter dorsal perineal artery, present in 106 of the hemi-pelvises studied. The study identified two major anatomic variations: one where the dorsal perineal artery was longer than usual and the ventral perineal artery was reduced (Figure 1b) that was present in 29 of the 232 hemi-pelvises studied, and another with an additional vessel called the “perineal trunk” (Figure 1c) where the dorsal perineal artery did not take origin directly from the caudal gluteal artery but from a common trunk. The long-type dorsal perineal artery originates from the caudal gluteal artery, vascularises both the dorsal and ventral parts of the perineum, and anastomoses with the ventral perineal artery, which is smaller in length and diameter. Therefore, the long dorsal perineal artery passes ventrally beyond the origin of the ventral perineal artery. In contrast, the short dorsal perineal artery only vascularises the dorsal part of the perineum without passing distally beyond the point of origin of the ventral perineal artery. If there was a dorsal perineal artery, either long or short, there was always a ventral perineal artery (normal or small, depending on whether the dorsal perineal artery was long or short). If there is a perineal trunk, there is no ventral perineal artery. The morphology of the perineal trunk can be of two types: a common trunk from which two branches clearly emerge, one corresponding to the dorsal perineal artery (which vascularises the dorsal part of the perineum) and the other corresponding to the ventral perineal artery (which vascularises the ventral part of the perineum), which may even occasionally emerge from the caudal rectal artery; and, on the other hand, in the trunk of the perineum, these two branches may not be clearly differentiated, and instead there may be several branches that emerge along their entire length in a dorsoventral direction to vascularise the entire perineum. A symmetry of 0.4% was observed between the right and left sides (Table 2).

It is interesting to note the gender difference in the variation of the perineal trunk: of the 41% of cases in which a perineal trunk was present, 24% were in males and 58% in females. In 76% (72 of the 95 pelvic halves with the perineal trunk), the perineal trunk gives off two branches, one proximal and one distal, corresponding to the dorsal and ventral perineal arteries. However, in 24% (23 of the 95 pelvic halves with the perineal trunk), the perineal trunk runs caudoventrally and gives off small, scattered branches along its course. There were six cases in the male pelvic halves and seventeen cases in the female pelvic halves. It is worth noting that the specific percentages for vascular symmetry patterns were particularly low for the dorsal perineal artery long-type vascular variation (0.4%) and also for the presence of the perineal trunk (5%) as well as in the presumed model (6%).

Two more isolated cases in females (right side) show particular anatomical variations. In one of them, the ventral perineal artery arises from the caudal gluteal artery distally to the origin of the cranial gluteal artery. In this case, the ventral perineal artery arises at the level of the sacrocaudal joint, courses caudoventrally, gives rise to the caudal rectal artery, and supplies the ventral part of the perineum, and the caudal rectal artery gives rise to the urethral artery. In the other, at the level of the second caudal vertebra, the internal pudendal artery gives rise to a common trunk for the lateral caudal artery, and a perineal trunk gives rise to the dorsal perineal and ventral perineal arteries. These identified vascular variations, which represent 0.8% of the cases studied, were not included in this study.

**Figure 2:**
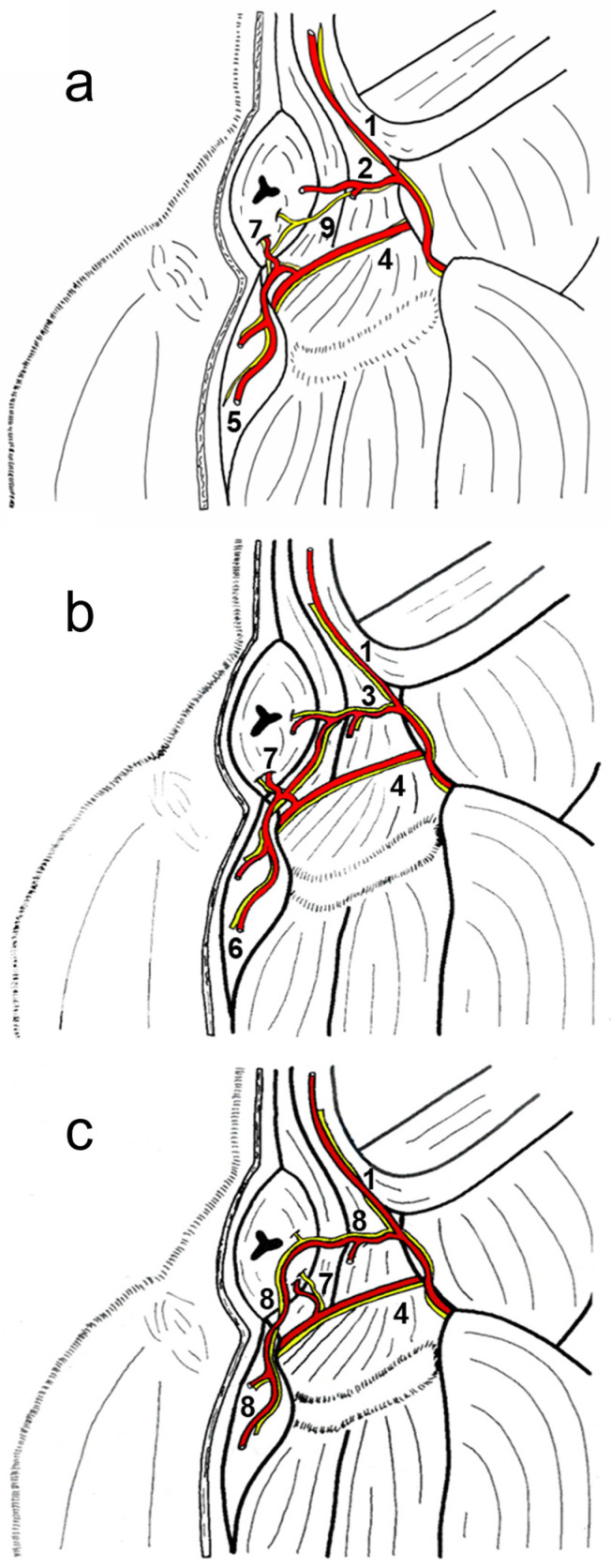
Schematic representation of the anatomical variations of the perineal arteries and the perineal innervation in the dog (caudal view). (**a**) NAV vascular pattern; (**b**) dorsal perineal artery long type; and (**c**) perineal trunk. 1: Lateral caudal artery; 2: Dorsal perineal artery short type; 3: Dorsal perineal artery long type; 4: Internal pudendal artery and pudendal nerve; 5: Ventral perineal artery; 6: Minor ventral perineal artery; 7: Caudal rectal artery and nerve; 8: Perineal trunk and superficial perineal nerve; 9: Superficial perineal nerve.

**Figure 3:**
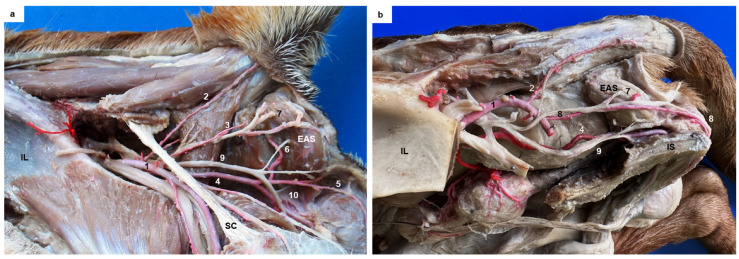
(**a**) Lateral view of the ischiorectal fossa of a dog presenting dorsal perineal artery short type. (**b**) Lateral view of the pelvic cavity of a dog presenting perineal trunk. 1: Caudal gluteal artery; 2: Lateral caudal artery; 3: Dorsal perineal, short type; 4: Internal pudendal artery; 5: Ventral perineal artery; 6: Caudal rectal artery and nerve; 7: Superficial perineal nerve; 8: Perineal trunk and superficial perineal nerve; 9: Pudendal nerve; 10: Deep perineal nerve; EAS: External anal sphincter; IL: Os ilium; IS: Os ischium; SC: Sacrotuberous ligament.

This study also examined the anatomical structures related to the surgical approach of the ischiorectal fossa and the perineal region in six dogs presenting the different vascular patterns. In addition, the nerves reaching the external anal sphincter were exposed in all cases. A schematic representation of the three anatomic variations in the perineal region is presented in Figure 2a–c. The external anal sphincter received its innervation via the caudal rectal nerve and deep and superficial perineal nerves (Figure 2a), all of which were exposed in all cases. The internal pudendal artery, vein, and nerve coursed caudomedially over the dorsal surface of the internal obturator muscle. The pudendal nerve gave off the caudal rectal nerve that ran together with the caudal rectal artery, and the deep perineal nerve ran together with the ventral perineal artery, both of which innervated the external anal sphincter (Figure 3a). The pudendal nerve continued ventrally as the dorsal nerve of the penis, and the superficial perineal nerve coursed dorsally, innervating the external anal sphincter and the skin (Figure 3a), and coursed together with the dorsal perineal artery long type and with the perineal trunk (Figure 3b). When the dorsal perineal artery was shorter, the superficial perineal nerve continued alone to innervate the external anal sphincter and the skin (Figure 3a).

## 4. Discussion

The traditional understanding of the origin and distribution of the dorsal and ventral perineal arteries is discussed. The dorsal perineal artery is said to arise from the caudal gluteal artery within the ischiorectal fossa and supply blood to the skin and adipose tissue of the perineal region, while the ventral perineal artery arises from the internal artery and supplies the ventral part of the perineal region. A wide variety of vascular variations have been described for the IIA of male and female dogs including vascular variations for the perineal arteries [1,2,3]. However, detailed information about the corresponding innervation of these arteries is limited. Despite the variations observed in the origin of the dorsal perineal and ventral perineal arteries described previously, we focus our discussion on the two vascular variations of the perineal arteries with the highest incidence and clinical relevance and the innervation of the perineal region including the ischiorectal fossa.

The classic vascular model for perineal arteries, called the dorsal perineal artery short type, was present in 45.7% of the studied cases. This suggests that this is the most common type of perineal artery variation in dogs. The study also found that the long-type dorsal perineal artery, which also corresponds to the presence of the short-type ventral perineal artery, was present in 12.5% of the cases studied (15% in male and 10% in female). The absence of the ventral perineal artery was observed in some cases with the perineal trunk present instead to vascularise the skin and the subcutaneous and adipose tissues within the perineal region. The perineal trunk was present in 41% of the studied cases. These findings suggest that variations in the perineal arteries are common in dogs, and surgeons should be aware of these variations when planning surgical approaches to this region. The perineal trunk was present in 95 of the hemi-pelvises studied. These variations were found to constantly course together with the superficial perineal nerve and supply the skin and the subcutaneous and adipose tissues of the perineal region. The presence of the perineal trunk was always related to the absence of the ventral perineal artery from the internal pudendal artery; in such cases, the tissue supplied by it was compensated by the dorsal perineal artery but also and mainly by the perineal trunk per se coming from the caudal gluteal artery.

A detailed knowledge of the perineal nerves and vessels is very important for veterinary surgeons when operating on the perineal area. Veterinary anatomy textbooks do not describe the vascular variations in detail. Therefore, this study can provide useful information for perineal surgery. The study of the nervous system in relation to the vascular system in the perineal area of dogs is valuable because of the postoperative complications on the nervous system that can occur in perineal surgery. As with the nervous system, the anatomical variations are also important in reducing intraoperative complications including bleeding in the perineal area. This study found that the pattern of innervation for the external anal sphincter and perineal region was constant with the external anal sphincter being innervated ventrally by the caudal rectal and deep perineal nerves and dorsally by the superficial perineal nerve, which ended by innervating the skin of the perineal region. The dorsal perineal artery long type and the perineal trunk were consistently found to course together with the superficial perineal nerve. This differs from the dorsal perineal artery short type described in the classic vascular model for perineal arteries, which does not follow the same course as the superficial perineal nerve. Understanding the anatomical variations in the perineal arteries and their relationship to the pattern of innervation of the perineal region and the external anal sphincter can be useful when planning surgical approaches to this region. The superficial perineal nerve ran together with the dorsal perineal artery in 53.5% of the studied cases. The general anatomical rule that an artery accompanies its companion nerve on the side from which it approaches was confirmed for the IIA and its branches as well as for the ventral perineal artery. However, the superficial perineal nerve only accompanied the dorsal perineal artery when it was of the long type or when the perineal trunk was present. If the nerves and vessels are not properly identified and preserved during dissection and surgery, they can be damaged, leading to potential complications. Therefore, understanding the variations in anatomy is crucial in minimizing the risk associated with surgical procedures; for instance, when major vessels are exposed and presumably are with their correspondent nerves, but they are not, nerves can be damaged during the dissection and surgery. It is essential to have a thorough understanding of the anatomical landmarks when planning any surgical procedure in the perineal region, particularly when it comes to reconstructive surgeries for perineal defects. Traditionally, the internal pudendal artery, vein, and pudendal nerve, as well as the caudal rectal nerve, have been considered important landmarks in this region [21,22]. The pudendal nerve is the primary nerve responsible for innervating the anal sphincter. The caudal rectal nerve, a branch of the pudendal nerve, provides motor and sensory innervation to the external anal sphincter. However, this study shows that the external sphincter is also innervated by the superficial and deep perineal nerves, challenging the notion that the caudal rectal vessels and nerves are the sole suppliers for this muscle [21,22,23]. Therefore, it is important to consider the contribution of all nerves and the possible arterial variations and regional blood supply in the perineal region when planning and performing surgical procedures. Additionally, perineal skin defects can be challenging to reconstruct due to the limited availability of adjacent skin for local mobilization and the need for large subdermal plexus flaps to cover extensive surface areas [6]. The complex and variable blood supply to the perineal region can make it difficult to assure adequate perfusion to a flap during reconstruction surgery when compared with similar flaps to other body regions [24,25]. Variations in the survival rate of flaps from different areas of the perineum, such as the scrotum [10,26], vulvar fold [11], and the rectus abdominus muscle [15], have been attributed to the variability in the vascular supply of the region. Therefore, a thorough understanding of the anatomy and variations in the blood supply to the perineal region is crucial when planning surgical procedures involving skin flaps. Variations in the number and location of branches of the cutaneous arteries can significantly affect the blood supply and viability of the flap and, hence, influence total flap circulation and viability. Distal necrosis is frequently found when axial pattern skin flaps are used in dogs [27]. The orientation of the flap pedicle must be carefully considered to ensure adequate perfusion pressure to the subdermal plexus. With a better understanding of the vascular supply of the perineal region, surgeons can design new flaps or modify existing ones to improve their success rates and minimize the risk of complications such as partial flap necrosis.

Postoperative haemorrhage is a potential complication that can occur after any surgical operation. In procedures in dogs such as perineal fistula and herniorrhaphy [7,28], anal sacculectomy [6,7], and cryosurgery [8], the perineal or caudal rectal arteries may be damaged, leading to bleeding [8,9]. The surgeon should have a thorough knowledge of the anatomy of the area being operated on, including the vascular variations, to minimize the risk of injury to nerves and vessels. This is particularly important in the perineal region, knowing that the superficial perineal nerve does not always course together with the dorsal perineal artery. Extreme care should be taken not to place sutures in or around the pudendal and/or perineal vessels and nerves nor to alter their course in any way. Proper suture technique and careful handling of tissues during surgery can help to minimize the risk of post-operative bleeding and other complications.

It is important for the surgeon to have a good understanding of the anatomical structures in the ischiorectal fossa to avoid injury to these structures during surgery. The internal pudendal artery and vein, pudendal nerve, caudal rectal vessels and nerve, external anal sphincter, rectum, sacrotuberous ligament, and the coccygeal, internal obturator, and elevator ani muscles are referred traditionally as important landmarks in the region [29]. During surgical procedures via the ischiorectal fossa, the surgeon may encounter the superficial and deep perineal nerves and caudal rectal nerve. These nerves are important for the innervation of the perineum and anal region and must be identified and preserved during tissue dissection. The vessels and nerves are contained within the adipose tissue, making them difficult to locate and identify. Therefore, extreme care should be taken during surgery to avoid injury to these important structures.

Most textbooks and authors consider that, within the ischiorectal fossa, the caudal rectal nerve, a branch of the pudendal nerve, is the sole innervation to the external anal sphincter [7,21,22,30]. This finding suggests that in some cases, the dorsal perineal artery may need to be considered as a potential risk during surgical procedures involving the ischiorectal fossa. The proximity or lack of proximity of this artery to the perineal superficial nerve, depending on the vascular pattern present, may increase the risk of accidental injury during dissection. It is important to evaluate the possible presence of vascular variations to minimize such risks. In this study, in 45.7% of the studied cases, the dorsal perineal artery did not run together with the perineal superficial nerve, and this should be considered when approaching the ischiorectal fossa. Surgical accidental and iatrogenic trauma can occur if a surgeon considers the location of the perineal nerve as described in surgery textbooks. Misidentification of the anatomical structures in the ischiorectal fossa may lead to damage of the superficial perineal nerve, which can partially compromise the innervation of the external anal sphincter. Even more, an excessive surgical dissection together with a wrong assumption for the location of the superficial perineal nerve can lead to partial anal denervation and its clinical presentation. Many surgical procedures may cause faecal incontinence, more often than what appears in surgical publications. Damage to the caudal rectal nerves or the sphincter muscle [21,31] as well as damage to perineal nerves can all lead to faecal incontinence.

Understanding the precise anatomical course and relations of the terminal innervation and vascularization of the external anal sphincter and the perineum is necessary for surgeons to minimize the risk of iatrogenic complications during surgical procedures. Surgeons should take into consideration the variations in the location of nerves and vessels when establishing identifiable landmarks to avoid complications during surgery. As an example, it is not ideal to use the dorsal perineal artery as a landmark to locate the superficial perineal nerve when the dorsal perineal artery is the short type. In addition, this knowledge can help surgeons develop new surgical approaches to the perineal region.

## 5. Conclusions

Understanding the neurovascular anatomy of the perineal region is crucial for the development of safe and effective surgical techniques. The surgeon should be aware of the high incidence of vascular variations within the pelvic cavity and the perineal region in dogs and how they may impact the innervation of the region. Additionally, the finding that the perineal nerve does not always run with the dorsal perineal artery and instead may run with the perineal trunk and with the dorsal perineal artery long type highlights the need for careful dissection and consideration of individual variations in each patient. The knowledge of the anatomical variations of the perineal arteries and their relationship to nerve disposition may help surgeons plan and execute surgical procedures more effectively and minimize the risk of iatrogenic complications such as faecal incontinence. The anatomical information contained herein is of interest to anatomists, surgeons, and anatomy teachers and students. For the latter, it is an added value, as it is important for them to understand how different anatomical regions can be from anatomy books.

## Figures and Tables

**Table 1:** Chi-squared data for gender, side, profile, and height; *p* ≤ 0.05 significant.

Degrees of Freedom	T Number	Gender 1	Side 1	Profile 2	Height 2
1.1. Pearson chi-square	1.1	22.5121	0.0695	7.4626	5.2221
*p*-value	1.1	0.0000	0.7921	0.0240	0.0735
1.2. Pearson chi-square	1.2	0.9852	1.9310	0.0577	4.8327
*p*-value	1.2	0.3209	0.1646	0.9716	0.0892
1.3. Pearson chi-square	1.3	27.1127	1.4439	7.4843	1.2365
*p*-value	1.3	0.0000	0.2295	0.0237	0.5389

**Table 2:** Data for vascular symmetry patterns in bilateral hemi-pelvises for each type of variation.

	Traditional Anatomical Model	Dorsal Perineal Artery “Long Type”	Perineal Trunk
*n*	11♂/4♀	1♂	3♂/9♀
%	6.46	0.43	5.17

*n*: Number of cases; %, percentages relative to the total number of samples.
